# miR-17-92 facilitates neuronal differentiation of transplanted neural stem/precursor cells under neuroinflammatory conditions

**DOI:** 10.1186/s12974-016-0685-5

**Published:** 2016-08-27

**Authors:** Susu Mao, Xiuhua Li, Jin Wang, Xin Ding, Chenyu Zhang, Liang Li

**Affiliations:** 1 State Key Laboratory of Pharmaceutical Biotechnology, Collaborative Innovation Center of Chemistry for Life Sciences, Jiangsu Engineering Research Center for MicroRNA Biology and Biotechnology, NJU Advanced Institute for Life Sciences (NAILS), School of Life Sciences, Nanjing University, 163 Xianlin Road, Nanjing, Jiangsu 210023 China; 2 Jiangsu Key Laboratory of Neuroregeneration, Co-Innovation Center of Neuroregeneration, Nantong University, Nantong, 226001 China

**Keywords:** miR-17-92, Neural stem/precursor cells, Transplantation, Neuroinflammation, JAK-STAT pathway, Differentiation

## Abstract

**Background:**

Neural stem/precursor cells (NSCs) are of particular interest because of their potential application in cell therapy for brain damage. However, most brain injury cases are followed with neuroinflammatory stress, which affects the lineage selection of grafted NSCs by promoting astrocytogenesis, thus hampering the potential for neural replacement. The present study investigated the role of miR-17-92 in protecting against detrimental effects of neuroinflammation on NSC differentiation in cell therapy.

**Methods:**

NSCs were treated with conditioned medium from lesioned astrocytes with/without neutralizing antibodies of leukemia inhibitory factor (LIF) or/and ciliary neurotrophic factor (CNTF), respectively. Afterward, the levels of p-STAT3 and p-JAK2 were determined by western blotting while expression of glial fibrillary acidic protein (GFAP) and β-tubulin III was assessed by immunostaining. The activation of JAK-STAT pathway and cell differentiation were also evaluated after we overexpressed miR-17-92 in NSCs under different neuroinflammatory conditions. After the transplantation of miR-17-92-overexpressing NSCs into injured mouse cortex, PH3, nestin, GFAP, and NeuN were analyzed by immunostaining. In addition, motor coordination of mice was evaluated by rotarod test.

**Results:**

Conditioned medium from lesioned astrocytes activated JAK-STAT pathway and facilitated astrocytic differentiation in NSCs while neutralizing antibodies of LIF and CNTF remarkably attenuated such effects. miR-17-92 cluster repressed the expression of multiple proteins including GP130, CNTFR, JAK2, and STAT3 in JAK-STAT pathway. Overexpression of miR-17-92 in NSCs systematically blocked the activation of JAK-STAT pathway mediated by LIF and CNTF, which facilitated neuronal differentiation in vitro. Furthermore, miR-17-92 increased neuronal generation of grafted NSCs and reduced astrogliosis, which resulted in the improvement of motor coordination of brain-injured mice.

**Conclusions:**

Our results suggest that miR-17-92 promotes neuronal differentiation of grafted NSCs under neuroinflammatory condition via inhibition of multiple proteins in JAK-STAT pathway.

**Electronic supplementary material:**

The online version of this article (doi:10.1186/s12974-016-0685-5) contains supplementary material, which is available to authorized users.

## Background

Neural stem/precursor cells (NSCs) consist of a heterogeneous population of self-renewing progenitor/precursor cells that can be expanded in vitro, which further give rise to neurons, astrocytes, and oligodendrocytes after transplanting into the central nervous system (CNS) [1, 2]. Recent studies suggest that transplantation of NSCs has become a promising therapy for various neurological disorders including ischemia, traumatic brain injury, and several neurodegenerative diseases of CNS [3, 4]. The tightly regulated cellular processes, especially the neural differentiation, are essential for grafted NSCs to produce enough neurons to restore neural damage in CNS [5]. The differentiation of NSCs is the result of changes in stem-cell properties that are controlled by both extrinsic and intrinsic cues [6]. Proneural genes are intrinsic determinants that control neurogenesis while astrocytogenesis can be initiated by several extrinsic gliogenic signals, such as ciliary neurotrophic factor (CNTF), leukemia inhibitory factor (LIF), and bone morphogenetic proteins (BMPs) [6, 7]. Substantial evidences suggest that neurological disease or injury induces a neuroinflammatory response in the CNS, which has great impact on endogenous neurogenesis as well as differentiation of grafted NSCs [8, 9]. Ideguchi et al. showed that inflammatory response by the host brain suppresses neuronal differentiation of transplanted ES cell-derived neural precursor cells [10]. The low yield of NSCs-derived neurons limits the potential clinical application for neuronal replacement.

In the condition of neuroinflammatory response, glia cells are activated which have been proved to affect neuronal survival [8]. In addition, it is also suggested that activated astrocyte can modulate NSCs differentiation [11]. As one of the key players mediating inflammatory response, astrocytes may produce several cytokines including LIF, CNTF, and interleukin 6 (IL-6) that have been proved to modulate neural differentiation in vitro [12]. Particular attention has been paid to LIF and CNTF because of their roles in the developmental switch from neurogenesis to astrocytogenesis [13]. LIF and CNTF promote premature generation of astrocytes in vitro via activation of the Janus kinase (JAK)-signal transducer and activator of transcription (STAT) pathway [14]. Furthermore, cytokine-induced STAT signaling directly activates JAK-STAT pathway in a positive, autoregulatory loop, resulting a potentiation of JAK-STAT-induced astrocytogenesis [15]. Therefore, we believe that the inhibition of JAK-STAT pathway in grafted NSCs under neuroinflammatory condition caused by brain injury may repress astrocytogenesis while enhancing the number of generated neurons, which may further exert beneficial effects on tissue regeneration and functional recovery.

MicroRNAs are approximately 21–22 nucleotides which modulate genes expression by targeting 3′ untranslated region (3′ UTR) of mRNAs [16]. Substantial evidences suggest that miRNAs are enriched in CNS indicating their significant role in neural development and physiology [17, 18]. Our previous work demonstrated that miR-17 modulates neuron-astrocyte transition via inhibiting BMPR2 expression [19]. Additionally, there are evidences that a cluster of miRNAs repress multiple targets coordinated in the same pathway, which show that those small RNAs have great potential in modulating cell signals [20–22].

In the present study, we demonstrated that LIF and CNTF secreted by astrocyte in neuroinflammatory condition facilitated astrocytogenesis of NSCs through activation of JAK-STAT pathway. We also showed that miR-17-92 cluster effectively blocked the activation of LIF/CNTF-JAK-STAT signal by repressing multiple protein targets existed in this pathway, leading to the increase of neurogenesis of grafted NSCs in vivo. Such improvement also benefited motor activity in injured mice after NSC transplantation.

## Methods

### Embryonic neurosphere culture and lentiviral transduction

Embryonic neurospheres (NSCs) were derived from the cortex of E14.5 wild-type C57BL/6J mice as previously described [19]. The cortex was dissected and mechanically dissociated, and the resulting single-cell suspension was placed in Dulbecco’s modified Eagle’s medium: nutrient mixture F-12 (DMEM/F12, Life Technologies, Grand Island, NY, USA) supplemented with N2 plus media supplement (R&D Systems, Minneapolis, MN, USA), 10 ng/ml recombinant bovine fibroblast growth factor (bFGF; R&D Systems), and recombinant human epidermal growth factor (hEGF; R&D Systems). After 5 days in culture, free-floating neurospheres were redissociated and allowed to reform spheres at least three times before further use. For differentiation studies, whole-mount or dissociated neurospheres were plated onto ploy-l-ornithine (Sigma-Aldrich, St. Louis, MO, USA) and human fibronectin (R&D Systems)-coated coverslips and further cultured in the absence of bFGF/hEGF.

The lentiviral vector lenti-17-92 used for miR-17-92 cluster overexpression under the control of the elongation factor 1-alpha promoter and the respective control vector driving EGFP expression were generated by GenePharma Inc. (Shanghai, China). For lentiviral tranduction, fourth-passage neurospheres were single-cell dissociated, grown in suspension for 24 h, and then exposed to lenti-17-92 or control lentivirus for 10 h. Transduction efficiency was estimated by quantitative real-time PCR (QPCR).

### Quantitative real-time PCR

Total RNA was isolated using TRIzol reagent (Life Technologies) according to the manufacturer’s instructions. Quantitative real-time PCR of mature miRNAs was performed using TaqMan microRNA probes (Applied Biosystems, Foster City, CA, USA) according to the manufacturer’s instructions. U6 snRNA was used for normalization in miRNA expression studies. All of the reactions were run in triplicates. A relative fold change in expression of miRNA was calculated with the equation 2^−ΔCT^.

### Astrocyte culture and preparation of conditioned medium

Primary astrocytes were purified from neonatal P0 mice cortices and plated in DMEM/F12/10 % FBS in poly-l-lysine (Sigma-Aldrich)-coated 75 cm^2^ culture flasks, as previously described [23]. Medium was changed after 3 days in culture and thereafter three times per week. After incubating for 2 weeks, to obtain pure astrocytes, the mixed cells were orbitally shaken at 200 rpm for 6 h, and the supernatant was removed. The attached cells were washed twice, digested by trypsin and then replated. When the astrocytes have reached 90 % of confluence at passage 3, cells were washed and a defined serum-free medium was added, containing DMEM/F12 and 1 % N2 plus supplement. After 6 h of equilibration in the serum-free medium, the cultures were mechanically lesioned with a pipette tip in a 2-cm-gird frame. Conditioned medium (CM) was collected 48 h after the injury, filtered at 0.22 μm and immediately stored at −80 °C until later use.

### Immunocytochemical and immunohistochemical procedures

Whole mount neurospheres and dissociated cells were fixed for 20 min in 4 % paraformaldehyde (PFA) followed by 1 h blocking with 5 % normal horse serum in phosphate-buffered saline (PBS)/0.3 % triton X-100. Incubation with primary antibodies was performed in 2 % BSA overnight at 4 °C. Cell nuclei were visualized with DAPI. For immunohistochemistry on tissue sections, mice were perfused transcardially with 50 ml PBS followed by 50 ml 4 % PFA. The brains were removed, post-fixed in the same fixative overnight at 4 °C, and cryoprotected in 15 % sucrose overnight followed by 30 % sucrose overnight. Cryostat sections (30 μm thick) were cut and processed for immunohistochemistry. Free-floating sections were incubated for 1 h in PBS/0.1 % triton X-100/5 % horse serum. The primary antibodies used on cells or tissue sections for multiple label immunofluorescence were as follows: rabbit polyclonal to glial fibrillary acidic protein (GFAP) (ab7779; 1:1000; Abcam, Hong Kong, China), chicken polyclonal anti-nestin (ab81755; 1:1000; Abcam), rabbit polyclonal anti-Sox2 (ab97959; 1:500; Abcam), mouse monoclonal to MAP2 (ab11267; 1:1000; Abcam), mouse monoclonal to CNPase (ab6319; 1:1000; Abcam), rabbit polyclonal anti-oligodendrocyte specific protein (ab53041; 1:1000; abcam), mouse monoclonal to neuron specific β-tubulin III (ab7751; 1:500; Abcam), mouse monoclonal anti-neuronal nuclei (NeuN) (MAB377; 1:100; Millipore, Billerica, MA, USA), rabbit polyclonal anti-p-histone H3 (sc-8656-R; 1:1000; Santa Cruz Biotechnology, Santa Cruz, CA), and rabbit polyclonal anti-Doublecortin (ab18723; 1:1000; Abcam). Following primary antibodies, the appropriate secondary antibodies were used: Alexa Fluor® 488-conjugated AffiniPure donkey anti-chicken IgY (IgG) (H+L) (703-545-155; 1:1000; Jackson ImmunoResearch, West Grove, PA, USA), Alexa Fluor® 594-conjugated AffiniPure donkey anti-chicken IgY (IgG) (H+L) (703-585-155; 1:1000; Jackson ImmunoResearch), Alexa Fluor® 488-conjugated donkey anti-mouse IgG (H+L) (A-21202; 1:1000; Life Technologies, Grand Island, NY, USA), Alexa Fluor® 594-conjugated donkey anti-rabbit IgG (H+L) (A-21207; 1:1000; Life Technologies), Alexa Fluor® 594-conjugated donkey anti-mouse IgG (H+L) (A-21203; 1:1000; Life Technologies). Finally, the cells or brain sections were stained with DAPI. For quantitative analysis, the numbers of each type of cell scored in four random fields were averaged, utilizing the Image-Pro Plus image analysis software.

### Western blot analysis

The cultured cells were lysed using RIPA buffer (Thermo Scientific, Rockford, IL, USA), and the protein was extracted according to the manufacturer’s instructions. Total proteins were determined using a Bicinchoninic Acid Protein Assay Kit (Thermo Scientific, Rockford, IL, USA). The samples were loaded onto 10 % SDS-polyacrylamide gel electrophoresis separating gel and transferred onto polyvinylidene difluoride membranes (Roche Diagnostics, Indianapolis, IN, USA). Primary antibodies used were as follows: goat polyclonal anti-p-JAK2 (sc-21870; 1:500; Santa Cruz), rabbit polyclonal anti-JAK2 (SC-294; 1:500; Santa Cruz), rabbit monoclonal anti-STAT3 (ab109085; 1:1000; Abcam), rabbit monoclonal anti-p-STAT3 (ab76315; 1:1000; Abcam), mouse monoclonal anti-CNTFR (sc-393214; 1:500; santa cruz), rabbit polyclonal anti-GP130 (3732; 1:1000; Cell Signaling Technology, Beverly, MA, USA), rabbit monoclonal to GFAP (ab68428, 1:2000; Abcam), and rabbit monoclonal anti-GAPDH (ab181602; 1:2000; Abcam). Horseradish peroxidase-conjugated anti-mouse, anti-rabbit, and anti-goat secondary antibodies (1:1000; Santa Cruz Biotechnology) were used. Color development was achieved by using the ECL Western Blotting Detection Kit (Thermo Scientific). Quantification of protein expression level was performed using the ImageJ analysis software.

### Luciferase assay

The CNTFR or GP130 3′ UTR containing the predicted target sequence was cloned and inserted into the pMIR-REPORT™ luciferase vector (Ambion, Austin, TX), respectively. The primers used were as follows. For CNTFR, forward primer: 5′CTAGACTAGTCACGAGGACATGCCAGAGC3′, reverse primer: 5′ CCCAAGCTTCCATTGAGTCAGACTAGAAGGGAC3′; for GP130, forward primer: 5′CTAGACTAGTCCTCACTCCCTGAAGATAGGC3′, reverse primer: 5′CCCAAGCTTGCCACCCTTCAACAAACACC3′. Three mutated vectors were generated by Life Technologies by replacing the predicted target region with its reverse sequence (mut CNTFR, from TTTGCAC to AAACGTG; mut1 GP130, from GCACTTT to CGTGAAA; mut2 GP130, from TTTGCAC to AAACGTG). HEK 293T cells were seeded onto 24-well plates for 12 h. Afterwards, 0.2 μg of firefly luciferase reporter plasmid; 0.2 μg of β-galactosidase expression vector (Ambion); 0.2 μg of expression vector pcDNA3.1-overexpressing miR-17-92 cluster; empty vector pcDNA3.1; or 10-, 20-, 50-nM miR-17, miR-20a, miR-19a, and miR-19b mimics were transfected into the cells. Cells were harvested for the luciferase assay (Promega, Madison, WI, USA) 24 h later, and the luciferase activity was normalized to the β-galactosidase activity.

### Traumatic brain injury and transplantation

All of the animal care and experimental procedures were performed in accordance with the Laboratory Animal Care Guidelines approved by the Model Animal Research Center of Nanjing University. Traumatic brain injury was performed as previously described [3]. Female C57BL/6 mice 10–12 weeks old were deeply anesthetized and positioned in a stereotaxic frame. A burr hole was drilled at coordinates AP 1.0, L 1.8 (relative to Bregma = 0) using a dental drill, and stab wound injury was caused to the right hemisphere of the cerebral cortex by inserting a 26-gauge needle 1.0 mm deep from the brain surface (DV 1.0 relative to Bregma = 0). The needle was then retracted and reinserted five times. Immediately after injury, 1 μl of freshly dissociated NSCs (10^5^ cells), either transduced with miR-17-92-overexpressing lentivirus or control lentivirus, was injected 0.3 mm ventrally to the injury site using a 1-μl syringe with a 26-gauge needle (SGE; Syringe perfection). Cells were injected slowly over 5 min, the syringe was left in place for an extra 5 min and then withdrawn gently, and the skin was sutured. After surgery, mice were held on a heated cushion before being returned to their home cages.

### Rotarod test

The rotarod test described by Hamm et al. was modified for use in mice [24]. Briefly, prior to surgeries, mice were trained on the rotarod for three consecutive days (three trials per day) at an accelerating speed increasing from 4 to 40 rpm over 5 min. To test for motor coordination, three trials for fast speed (32 rpm) were performed up to 5 min with 30-min intervals between trials and the best performance value for each animal was recorded. Mice that failed to stay on the rotarod for >30 s at 32 rpm were excluded from the experiment. A trial was terminated if the animal fell off the rotarod or gripped the device and spun around past the lowest point. Post-injury testing was monitored at 1, 4, 6, and 12 weeks. Three different groups of mice were tested: (1) mice with brain injury without cell transplantation (*n* = 8 mice), (2) mice with brain injury that received control grafts of CON-NSC (*n* = 8 mice), and (3) mice with brain injury that received grafts of miR-17-92-NSC (*n* = 8 mice). Mice were cared for in accordance with institutional guidelines.

### Statistical analysis

Data are presented as means ± SEM of at least three independent experiments. Direct comparisons were made using Student’s *t* tests, and multiple group comparisons were made using one-way analysis of variance (ANOVA) followed by Tukey’s test. Statistical significance was defined as *P* < 0.05, 0.01, or 0.001 (indicated as *, **, or ***, respectively). PRISM 5.0 software (GraphPad Inc., La Jolla, CA) was used for data analysis.

## Results

### Inhibition of LIF and CNTF secreted by reactive astrocyte reduced astrocytogenesis in neuroinflammatory condition

**Fig. 1:**
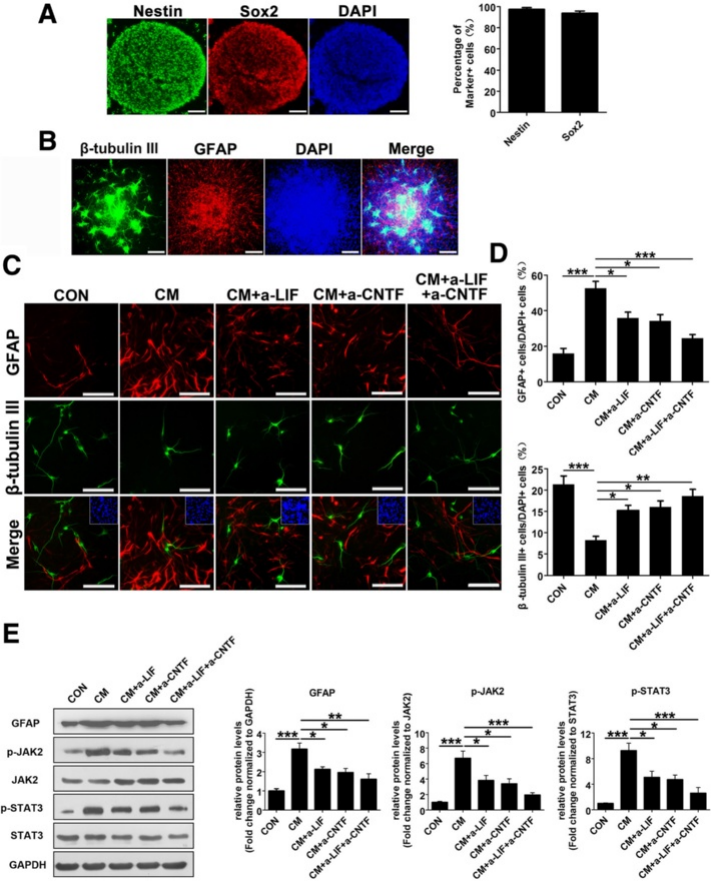
Effects of reactive astrocytes on NSC differentiation. **a** Immunofluorescence labeling of whole-mount embryonic cortical neurospheres showing the expression of NSC marker nestin (*green*); DAPI (*blue*) was used for nuclear staining. Scale bar, 100 μm. **b** The whole-mount neurospheres allowed to differentiate for 7 days in the absence of bFGF and hEGF were stained with the neuron marker β-tubulin III (*green*) and astrocyte marker GFAP (*red*); DAPI (*blue*) was used for nuclear staining. Scale bar, 200 μm. **c** The dissociated neurospheres were exposed to DMEM/F12/1 % N2 plus media supplement medium (CON) and reactive astrocyte-conditioned medium alone (CM), with LIF or/and CNTF neutralization antibody (CM + a-LIF, CM + a-CNTF, or CM + a-LIF + a-CNTF) to differentiate for 4 days in the absence of bFGF and hEGF, followed by fluorescence staining with β-tubulin III (*green*) and GFAP (*red*). *Inset*: DAPI staining of cell nuclei in the field of view. Scale bar, 100 μm. **d** The proportions of GFAP-positive or β-tubulin III-positive cells from the experiment shown in **c** were determined. Values are means ± SEM. *Asterisks* indicate a statistically significant difference compared with the CON or CM group (*P < 0.05, **P < 0.01, ***P < 0.001, one-way analysis of variance (ANOVA) followed by Tukey’s test, *n* = 3 independent experiments). **e** Western blot analysis and quantifications of p-JAK2, p-STAT3, and GFAP protein expressions in NSCs treated with different conditioned media indicated in **c** for 4 days. JAK2 and STAT3 were used as internal controls. Values are means ± SEM. *Asterisks* indicate a statistically significant difference compared with the CON or CM group (*P < 0.05, **P < 0.01, ***P < 0.001, one-way ANOVA followed by Tukey’s test, *n* = 4 independent experiments). For quantitative analysis, the numbers of each type of cell scored in four random fields were averaged, utilizing the Image-Pro Plus image analysis software

To investigate the effects of neuroinflammation on embryonic-derived NSCs differentiation, we first isolated NSCs from E14.5 C57BL/6 mouse cortex. Almost all NSCs were well stained with anti-nestin and Sox2 antibody in the sphere culture (97.4 ± 1.7 and 93.8 ± 2.0 %, respectively) (Fig. 1a), while DCX staining is relatively weak (Additional file 1: Figure S1A). These cultured spheres started to differentiate after withdrawing bFGF and hEGF, in which early neural marker β-tubulin III, mature neural marker MAP2, astrocytic marker GFAP, oligodendrocyte marker CNPase and oligodendrocyte-specific protein (OSP) were observed (Fig. 1b, Additional file 1: Figure S1B, C). Next, NSCs were cultured in astrocytic-conditioned medium (hereafter refers to as CM) from lesioned astrocytes which mimic neuroinflammatory condition in vitro. Compared to control group, NSCs are subjected to CM treatment differentiated into more astrocytes and less neurons (Fig. 1c, d). Since previous study has reported that LIF and CNTF are upregulated in conditioned medium from reactive astrocytes [25], we first confirmed that LIF and CNTF stimulation activated JAK-STAT pathway and significantly increased the ratio of GFAP-positive to β-tubulin III-positive cells in the NSC population under differentiation conditions (Additional file 1: Figure S2). To investigate the role of endogenous LIF and CNTF in CM on NSCs differentiation, we employed LIF or/and CNTF-neutralizing antibodies in CM, which remarkably decreased the proportion of GFAP-positive cells, while increasing that of β-tubulin III-positive cells compared to the CM group (Fig. 1c, d). Additionally, we further evaluated the activation status of JAK2/STAT3 signaling pathway by western blot analysis. We found that CM stimulation significantly increased the levels of p-JAK2, p-STAT3, and GFAP in NSCs, while LIF or/and CNTF-neutralizing antibody treatment blocked such upregulation in those cells (Fig. 1e). These findings suggest that LIF and CNTF secreted by reactive astrocytes activate JAK2/STAT3 pathway, which further induce astrocytogenesis and inhibit neurogenesis of NSCs while inhibiting these signals can significantly reduce astrocytogenesis of NSCs in neuroinflammatory condition.

### miR-17-92 cluster targets multiple proteins in JAK2/STAT3 pathway mediated by LIF/CNTF

**Fig. 2:**
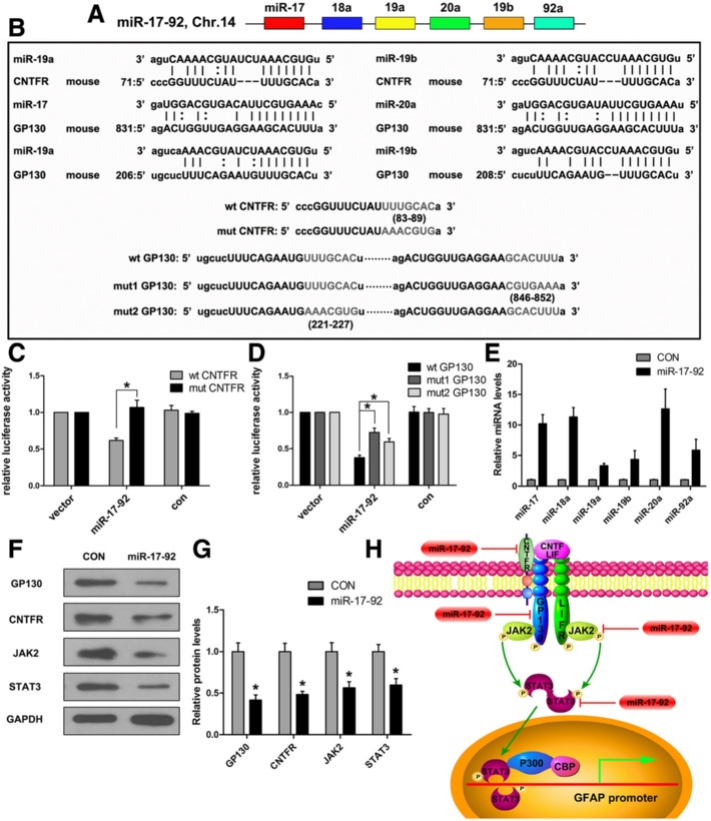
miR-17-92 cluster members target multiple proteins in JAK2/STAT3 pathway. **a** The schematic genomic organization of the miR-17-92 cluster on the mouse chromosome 14 (Chr.14). **b** Sequence alignment of mature miR-19a, miR-19b, miR-17, and miR-20a revealed their seed sequences that were reverse complementary to the seed-matched sequence within the 3′ UTR of mouse CNTFR or GP130, respectively. The mutated sequences in the 3′ UTRs of CNTFR (mut CNTFR) and GP130 (mut1 GP130, mut2 GP130) are indicated. **c**, **d** Luciferase activity was measured 24 h after transfecting HEK 293T cells. Reporter plasmids with the wild-type (wt CNTFR, wt GP130) or mutated (mut CNTFR, mut1 GP130, or mut2 GP130) 3′UTR of CNTFR or GP130 were transfected either alone (vector) or with the expression vector pcDNA3.1-overexpressing miR-17-92 cluster (miR-17-92) or empty pcDNA 3.1 (con). Values are means ± SEM. *Asterisks* indicate a statistically significant difference compared with the control (*P < 0.05, one-way ANOVA followed by Tukey’s test, *n* = 3 independent experiments). **e** Quantitative real-time PCR was used to determine the efficiency of overexpressing miR-17-92 cluster members using lentivirus (miR-17-92). Values are means ± SEM (*n* = 3 independent experiments). **f** Western blot analysis of GP130, CNTFR, JAK2, and STAT3 protein expressions in NSCs 72 h after infecting them with lentivirus overexpressing miR-17-92 cluster (miR-17-92) or control lentivirus (CON). **g** Quantifications for the protein expression levels from the experiment shown in **f**. Values are means ± SEM. *Asterisks* indicate a statistically significant difference compared with the control group (*P < 0.05, unpaired two-tailed *t* test, *n* = 3 independent experiments). **h** Schematic representation of multiple proteins regulated by miR-17-92 cluster in JAK2/STAT3 signaling pathway

In our previous study, we demonstrated that miR-17 modulates the astrocytogenesis/neurogenesis transition during the mouse corticogenesis by targeting BMP signaling pathway [19]. In addition, miR-17 and miR-20a are reported to target JAK2 and STAT3 [26, 27]. Furthermore, using TargetScan (http://Targetscan.org) and microRNA.org (http://microRNA.org) bioinformatics tools, we found that among those miRNAs in miR-17-92 cluster, miR-19a/b may target CNTFR and miR-17, miR-20a, and miR-19a/b may target glycoprotein 130 (GP130), respectively (Fig. 2a, b). Luciferase assay revealed that miR-17-92 cluster members bind to 3′UTR of CNTFR and GP130 directly (Fig. 2c, d; Additional file 1: Figure S3). When we overexpressed miR-17-92 cluster in NSCs, we found that the levels of miR-17-92 cluster members were all enhanced in various degrees (Fig. 2e), while the levels of targeted proteins including GP130, CNTFR, JAK2, and STAT3 were all declined (Fig. 2f, g). Moreover, we found similar miRNA-binding sites exited in these four genes in human database indicating these miRNA gene regulations may also exist in humans (Additional file 1: Figure S4). Based on these results, it is believed that miR-17-92 cluster can systematically regulate LIF/CNTF and downstream JAK2/STAT3 signaling by targeting multiple proteins (GP130, CNTFR, JAK2, and STAT3) in this pathway (Fig. 2h).

### miR-17-92 cluster represses JAK2/STAT3 signaling pathway and modulates neurogenesis/astrocytogenesis transition of cultured NSCs

**Fig. 3:**
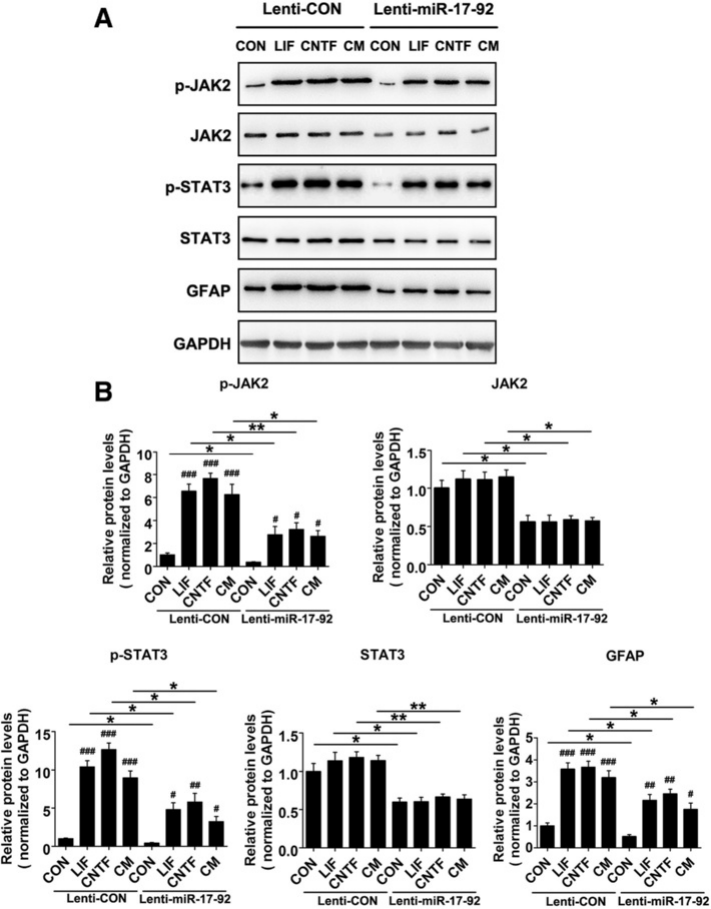
miR-17-92 cluster modulates JAK2-STAT3 signaling pathway. **a** Seventy-two hours after infecting them with lentivirus overexpressing miR-17-92 cluster (Lenti-miR-17-92) or control lentivirus (Lenti-CON), NSCs were cultured in the different differential media (DMEM/F12/1 % N2 plus media supplement medium alone (CON) or with 10 ng/ml LIF, CNTF or CM) for 4 days. Expression levels of p-JAK2, JAK2, p-STAT3, STAT3, and GFAP were analyzed by western blot. GAPDH was used as an internal control. **b** Quantification of the protein expression levels from the experiment shown in **a**. Values are means ± SEM. *Asterisks* indicate a statistically significant difference compared with the Lenti-CON group (*P < 0.05, **P < 0.01, unpaired two-tailed *t* test, *n* = 3 independent experiments). *Pound signs* indicate a statistically significant difference compared with the untreated control in the same group (^#^ *P* < 0.05, ^##^ *P* < 0.01, ^###^ *P* < 0.001, one-way ANOVA followed by Tukey’s test, *n* = 3 independent experiments)

To investigate the role of miR-17-92 cluster in modulating JAK2/STAT3 pathway mediated by LIF/CNTF, we overexpressed miR-17-92 cluster in cultured NSCs followed with LIF, CNTF, or CM treatment. Afterwards, we examined the protein levels of JAK2, STAT3, phosphorylated JAK2, phosphorylated STAT3, and GFAP (Fig. 3a). Our results demonstrated that miR-17-92 cluster significantly reduced the expression levels of JAK2 and STAT3, as well as their phosphorylation levels and their downstream GFAP protein levels (Fig. 3b). These data suggest miR-17-92 cluster overexpression in NSCs represses JAK2/STAT3 signaling pathway activated by the secreted LIF/CNTF during neuroinflammation.

**Fig. 4:**
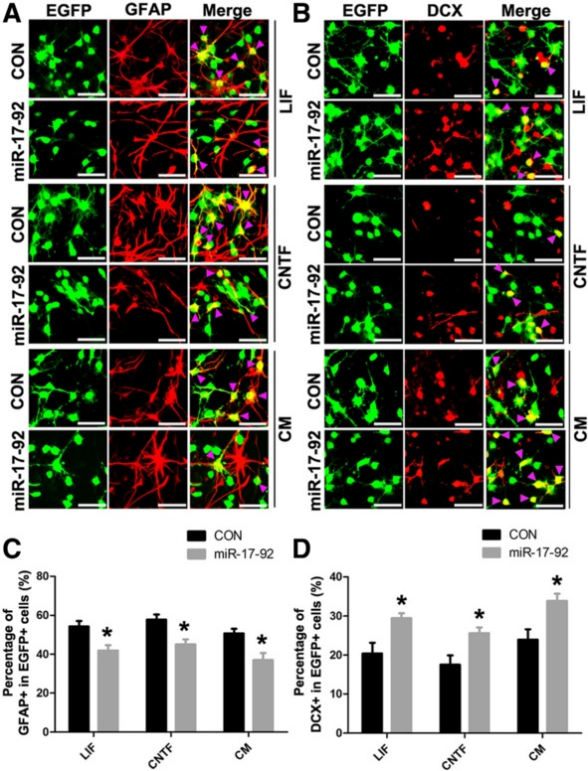
miR-17-92 cluster regulates astrocytogenesis and neurogenesis of NSCs in vitro. **a**, **b** Immunofluorescence labeling of NSCs showing the expression of astrocyte marker GFAP or neuron marker doublecortin (DCX) after infecting with lentivirus overexpressing miR-17-92 cluster (miR-17-92) or lentivirus expressing EGFP alone (CON) in the different differential media. *Arrow heads* indicate double positive cells. *Scale bar*, 50 μm. **c**, **d** Percentage of GFAP-positive or DCX-positive among total EGFP-positive cells from the experiment shown in **a** or **b** was determined. Values are means ± SEM. *Asterisks* indicate a statistically significant difference compared with the control group (*P < 0.05, unpaired two-tailed *t* test, *n* = 3 independent experiments)

To evaluate the effects of miR-17-92 cluster on NSC differentiation, we overexpressed miR-17-92 cluster in NSCs before LIF, CNTF, or CM stimulation, respectively. Afterwards, we assessed the level of astrocytogenesis and neurogenesis of differentiated NSCs. As compared to the control group, overexpression of miR-17-92 cluster decreased the percentage of GFAP-positive cells in the total EGFP-positive cells (41.9 ± 2.8 vs.54.3 ± 2.7 %, *p* < 0.05; 45.0 ± 2.6 vs. 57.8 ± 2.6 %, *p* < 0.05; 37.0 ± 3.7 vs. 50.7 ± 2.4 %, *p* < 0.05; Fig. 4a, c), while increasing the DCX-positive cell ratio in the total EGFP-positive cells (29.5 ± 1.3 vs. 20.4 ± 2.7 %, *p* < 0.05; 25.6 ± 1.5 vs. 17.5 ± 2.4 %, *p* < 0.05; 33.9 ± 1.8 vs. 23.9 ± 2.7 %, *p* < 0.05; Fig. 4b, d) in differentiated NSCs upon these stimulations.

Taken together, these results suggest that miR-17-92 cluster members can effectively repress LIF/CNTF-mediated JAK2/STAT3 signaling pathway by targeting GP130, CNTFR, JAK2, and STAT3, which further facilitates neuronal differentiation at the expense of astrocytogenesis of NSCs in vitro.

### miR-17-92 cluster represses astrocytogenesis and increases neurogenesis in grafted NSCs in vivo

**Fig. 5:**
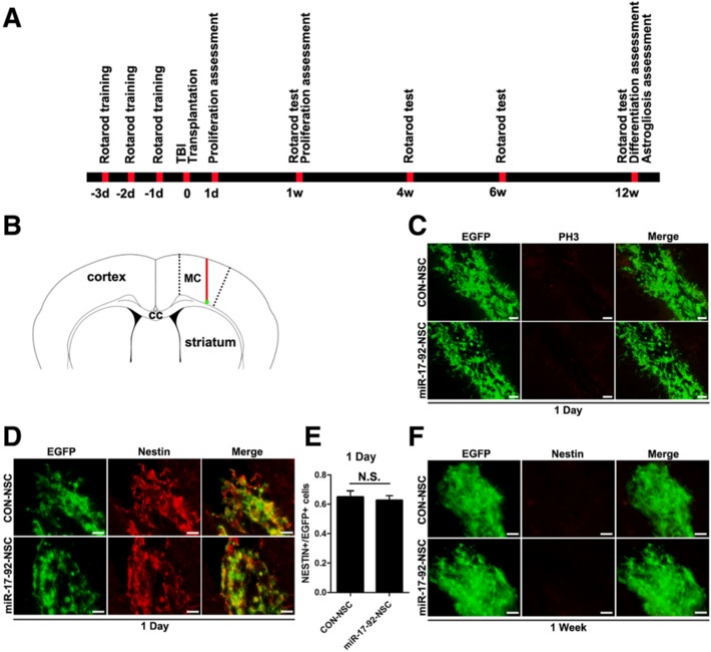
PH3 and nestin immunoreactivity in grafted NSCs. **a** Schematic presentation of temporal course of transplantation, immunohistochemical test, and behavioral analysis. **b** Diagram illustrating the injury site (*red line*) in the motor cortex (MC) and NSC grafting (*green dot*). **c** Immunofluorescence labeling of coronal sections for the mitotic marker PH3 (*red*), 1 day after transplantation of either control (CON-NSC) or miR-17-92-overexpressing (miR-17-92-NSC) cells (*green*). No positive signal was observed in the grafted cells. *Scale bar*, 50 μm. **d**, **f** Immunofluorescence labeling of coronal sections for the NSC marker nestin (*red*), 1 day and 1 week after transplantation of either CON-NSC or miR-17-92-NSC (*green*). Both types of grafted cells express nestin at 1 day, while no positive signal was observed at 7 days. *Scale bar*, 50 μm. **e** The ratio of nestin-positive to EGFP-positive cells from the experiment shown in **d** was determined. There was no statistically significant difference between the two groups (*N.S.* not significant, unpaired two-tailed *t* test, *n* = 3 independent experiments)

To further investigate the effects of miR-17-92 cluster on the differentiation of grafted NSCs in vivo, we first generated a stab wound injury in the motor cortex of adult C57BL/6 mice. We examined the status of astrocytes activation by staining GFAP around the injured area. We found that 1 day after injury, there were already an amount of reactive astrocytes and the number of reactive astrocytes increased 1 week after the injury (Additional file 1: Figure S5A), indicating a progressive inflammatory condition. Four weeks after injury, the damaged area was basically healed where there were no neuron-specific nuclear protein NeuN-positive cells (Additional file 1: Figure S5B). The temporal course of transplantation, immunohistochemical test, and behavioral analysis were depicted in Fig. 5a. Immediately after injury, NSCs were grafted 0.3 mm ventrally to the injured area just above the subcortical white matter (Fig. 5b). There were no mitotic marker phospho-histone H3 (PH3)-positive signals in NSCs overexpressing miR-17-92 cluster (miR-17-92-NSC) as well as control cells (CON-NSC) 1 day (Fig. 5c) and 7 days (data not shown) after transplantation, excluding the possibility of tumor formation which also indicates that miR-17-92 cluster has little effect on NSC proliferation in the lesioned brain.

To explore whether grafted NSCs retained their neural stem/precursor cell identity, the expression of NSCs marker nestin was determined. One day after transplantation, both miR-17-92-NSC and CON-NSC groups highly expressed nestin (64.9 ± 4.2 vs. 62.6 ± 3.2 %, *p* = 0.68), and there were no nestin-positive cells 1 week after transplantation (Fig. 5d–f). These findings reveal that miR-17-92 cluster has little effect on grafted NSCs in retaining their neural stem cell identity in the injured area.

**Fig. 6:**
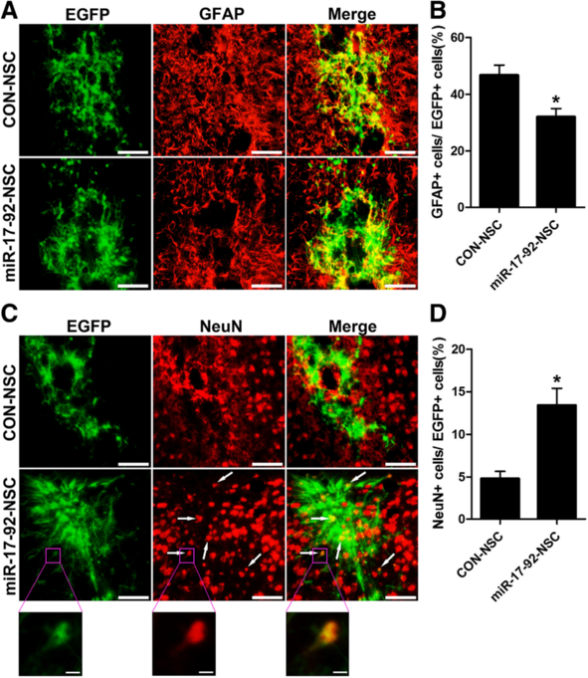
miR-17-92 cluster regulates astrocytogenesis and neurogenesis of grafted NSCs. **a**, **c** Expression of GFAP (*red*) or NeuN (*red*) in transplanted CON-NSC or miR-17-92-NSC cells (*green*), 12 weeks after injury and transplantation. *Arrows* in **c** indicate double positive cells. Scale bar, 100 μm. The framed areas in **c** are shown at higher magnification. Scale bar, 10 μm. **b**, **d** Percentage of GFAP-positive or NeuN-positive cells among total EGFP-positive cells from the experiment shown in **a** or **c** was determined. Values are means ± SEM. *Asterisks* indicate a statistically significant difference compared with the control group (*P < 0.05, unpaired two-tailed *t* test, *n* = 3 independent experiments)

To further evaluate the potential role of miR-17-92 cluster on NSC differentiation in injured brain under inflammatory condition, we examined the percentage of GFAP or NeuN-positive cells among grafted cells 12 weeks after transplantation. As compared to the control group, miR-17-92 cluster significantly decreased the proportion of astrocytes (32.1 ± 2.9 vs. 46.8 ± 3.5 %, *p* < 0.05; Fig. 6a, b), while that of neurons was remarkably increased (13.4 ± 2.0 vs. 4.8 ± 0.9 %, *p* < 0.05; Fig. 6c, d).

### miR-17-92 cluster overexpression reduces astrogliosis and improves the motor coordination of brain-injured mice

**Fig. 7:**
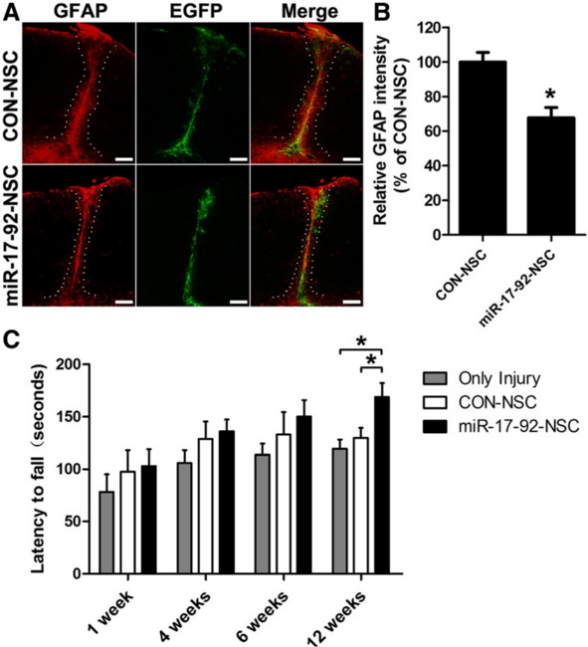
miR-17-92 cluster overexpression in grafted NSCs reduces astrogliosis and improves the motor coordination of brain-injured mice. **a** Immunostaining for GFAP (*red*), 12 weeks after transplantation of CON-NSC or miR-17-92-NSC (*green*). The area within *white dotted lines* corresponds to the astroglial scar. Scale bar, 200 μm. **b** Quantification of astrogliosis, as measured by relative GFAP fluorescence intensity from all coronal sections containing the injury site along the rostro-caudal axis (*P < 0.05, unpaired two-tailed *t* test, *n* = 3 independent experiments). **c** The mice that received control NSC (CON-NSC), miR-17-92-overexpressing NSC (miR-17-92-NSC), or no cells (Only Injury) were tested 1, 4, 6, and 12 weeks after injury and transplantation. *Asterisks* indicate a statistically significant difference compared with the only injury group or CON-NSC group (*P < 0.05, one-way ANOVA followed by Tukey’s test, *n* = 8 mice)

We observed that there are large amount of reactive astrocytes gathering around the lesion site, which normally reflects the recovery situation after brain injury. Therefore, we assessed the potential effect of miR-17-92 cluster overexpression on astrogliosis 12 weeks after NSC transplantation by immunostaining of GFAP. Our results showed that miR-17-92 cluster overexpression in NSCs significantly decreased astrogliosis by 32.2 % at the lesion site (Fig. 7a, b). This is partly because of the reduced astrocytogenesis of grafted NSCs, which directly diminished the number of reactive astrocytes. Meanwhile, the result also points to a potential non-cell autonomous effect of grafted NSCs on host astrocytes that contributes to the reduction of astrogliosis.

Due to the mild injury in the motor cortex, no obvious motor defects related to walking, climbing, or feeding abilities were observed among almost all the operated animals. Therefore, we checked the potential effect of miR-17-92-NSC transplantation using a rotarod test, a more sophisticated task of motor coordination. We showed that mice that received cell grafts of either type appeared to be doing better than the group that received no cells, especially the miR-17-92-NSC group. The mice that received NSC-overexpressing miR-17-92 cluster showed no significant differences in performance compared to control-NSC group until 12 weeks after transplantation (168.8 ± 13.6 vs. 129.8 ± 9.5, *p* < 0.05; Fig. 7c). These data indicate that miR-17-92 cluster may improve the motor coordination of brain-injured mice via increasing neurogenesis of grafted NSCs.

## Discussion

Transplantation of NSCs into the injured CNS becomes a promising strategy to overcome the regenerative limitations of the lesioned brain [28]. However, the pathological environment in the injured brain strongly affects grafted NSC properties and their lineage selection, which results in low yield of differentiated neurons [10, 29]. It is demonstrated that reactive astrocyte during CNS injury facilitates astrocytic differentiation of NSCs in vitro [25]. Those activated astrocyte under neuroinflammatory condition can secret several cytokines such as LIF and CNTF, which have been proved to regulate NSC differentiation in vitro [30]. In the present study, we have demonstrated that astrocytic CM strongly induces astrocytogenesis from NSCs and this induction is mitigated by the addition of neutralizing antibody of LIF and CNTF. These data indicate that LIF and CNTF are the major factors produced in astrocytic CM which promote glial cell fate of NSCs. In the nervous system, the effects of LIF and CNTF are complicated which depend on the stages of development. In the gliogenic phase, there are evidence that LIF and CNTF enhance generation, maturation, and survival of oligodendrocytes [31]. It is also reported that LIF is secreted by grafted NSCs and provides protective effects in animal model of multiple sclerosis by promoting survival, differentiation, and the remyelination capacity of both endogenous oligodendrocyte precursors and mature oligodendrocytes [32, 33]. However, in the early neurogenic phase when NSCs mainly give rise to neurons, LIF and CNTF can modulate neuron-astrocyte transition by promoting astrocytic differentiation from NSCs [34]. There is a similar, post-transplantation transition of neurons into astrocytes [35]. It is believed that inhibition of LIF and CNTF signals can reduce astrocytogenesis and enhance neuronal generation from NSCs [34].

There are circumstantial evidences suggesting that JAK-STAT pathway, which plays a crucial role in astrocytogenesis, is activated upon LIF, CNTF, and IL-6 stimulation [36, 37]. Furthermore, several studies suggest that inhibition of JAK-STAT pathway can induce fate switch and facilitate neuronal differentiation [38, 39]. We have demonstrated that astrocytic CM stimulation activates JAK-STAT pathway, while blocking LIF and CNTF in astrocytic CM significantly repress such activation leading to the increase of neuronal differentiation. These results together suggest that inhibition of LIF/CNTF signal and JAK-STAT pathway in NSCs under inflammatory conditions may increase neurogenesis at the expense of astrocytogenesis.

Our previous work demonstrated that miR-17 modulates neuron-astrocyte transition via inhibiting BMPR2 expression [19]. Meanwhile, Naka-Kaneda et al. also reported the role of miR-17 in controlling neurogenic to gliogenic transition of NSCs [40]. In the present work, we found that miR-17-92 cluster has multiple protein targets in LIF/CNTF signal pathway including CNTFR, GP130, JAK2, and STAT3. Among these four identified targets, JAK2 and STAT3 have been proved to be regulated by miR-17 in several models [26, 41, 42]. In addition, there are similar miRNA-binding sites in these four genes in the human database, indicating that such regulation of miR-17-92 cluster is conserved in humans. Further investigation showed that overexpression of miR-17-92 cluster significantly reduces those protein levels in NSCs leading to strong inhibition of JAK-STAT pathway under LIF/CNTF or astrocytic CM stimulation. As a result, such multi-targeted inhibition promoted neurogenesis while reducing astrocytic differentiation from NSCs subjected to LIF/CNTF or astrocytic CM treatment. These results demonstrated great potential of miR-17-92 cluster in promoting neuronal differentiation from NSCs under inflammatory condition in vitro.

Next, we assessed the effect of miR-17-92 cluster in modulating NSC differentiation under inflammatory conditions in vivo. Although it is reported that miR-17-92 cluster promotes cell expansion in vitro and in vivo [43, 44], in our study, overexpression of miR-17-92 cluster showed no significant impact on the proliferation of grafted NSCs since a majority of NSCs stop dividing 1 day after transplantation. Meanwhile, overexpression of miR-17-92 cluster significantly reduced astrocytogenesis and increased neuronal differentiation after NSCs transplantation in our brain injury model. Besides, we demonstrated that overexpression of miR-17-92 cluster in NSCs also reduced astrogliosis in the lesion site after cell transplantation. There are evidences that miRNA-containing microvesicles regulate inflammation in a cell-to-cell manner [45]. Additionally, miR-17-92 cluster is suggested as a key player in control inflammation [46]. Furthermore, several evidences suggest that miRNA-containing microvesicles are involved in cell-to-cell communication in CNS under pathological conditions related to neuroinflammation [23, 47]. Together, these data suggest that grafted NSCs may secrete microvesicles containing miR-17-92 cluster, which further modulate the inflammatory response of resident astrocytes that ultimately contributes to the reduction of astrogliosis.

Behavioral test of brain-injured mice showed remarkable improvement of motor coordination when miR-17-92 cluster-overexpressing NSCs were grafted, which is in agreement with our morphological findings. It is well known that increased neuronal differentiation is helpful to the functional recovery after CNS insults [3]. Besides, another potentially beneficial effect of miR-17-92 cluster-overexpressing NSCs is the significant inhibition of astrogliosis since reactive astrocyte as well as glial scar may have detrimental consequences after CNS damage [48, 49].

It is undoubted that during brain lesion, both astrocyte and microglia are activated, which result in a neuroinflammatory condition [50]. Cytokines secreted by both cell types may affect the cell lineage selection of grafted NSCs. In addition to the LIF and CNTF secreted by activated astrocyte, it is demonstrated that activated microglia also facilitates astrocytogenesis via producing IL-6 and LIF [8, 10]. Notably, all these cytokines affect NSCs differentiation through the modulation of JAK-STAT pathway [51, 52]. Furthermore, one of the targeted proteins of miR-17-92 cluster, GP130, controls IL-6, and downstream JAK-STAT pathway that regulates NSC lineage selection [31, 51]. Therefore, we believe that overexpression of miR-17-92 cluster in grafted NSCs can remarkably block the activation of JAK-STAT pathway mediated by LIF/CNTF/IL-6 under neuroinflammatory condition in vivo.

## Conclusions

Taken together, our results suggest that LIF and CNTF produced by reactive astrocyte activate JAK-STAT pathway in NSCs. miR-17-92 cluster effectively inhibits the activation of JAK-STAT pathway under neuroinflammatory condition via targeting multiple proteins, leading to the increase of neuronal differentiation from grafted NSCs after brain injury. These findings not only provide a better understanding of NSC differentiation regulated by neuroinflammation but also potentiate the clinical application of neural cell replacement.

## Additional file

Additional file 1: Figures S1–S5.
**Figure S1**. Characterization of the NSCs. **Figure S2**. Effect of LIF and CNTF on differentiation of NSCs. **Figure S3**. miR-17-92 members directly target the 3′UTR of CNTFR or GP130. **Figure S4**. Bioinformatics analysis of miR-17-92 cluster members binding sites within CNTFR, GP130, JAK2, and STAT3. **Figure S5**. Traumatic brain injury. (PDF 558 kb)

## Abbreviations

NSCs: Neural stem/precursor cells
LIF: Leukemia inhibitory factor
CNTF: Ciliary neurotrophic factor
GP130: Glycoprotein 130
CNTFR: Ciliary neurotrophic factor receptor
JAK2: Janus kinases 2
STAT3: Signal transducer and activator of transcription 3
CNS: Central nervous system
BMP: Bone morphogenetic protein
IL-6: Interleukin 6
3′UTR: 3′ Untranslated region
bFGF: Bovine fibroblast growth factor
hEGF: Human epidermal growth factor
GFAP: Glial fibrillary acidic protein
OSP: Oligodendrocyte-specific protein
CM: Conditioned medium
NeuN: Neuronal nuclei
PH3: Phospho-histone H3
DMEM/F12: Dulbecco’s modified Eagle’s medium: nutrient mixture F-12
DAPI: 4′,6-Diamidino-2-phenylindole
GAPDH: Glyceraldehyde 3-phosphate dehydrogenase
EGFP: Enhanced green fluorescent protein
