# Supplementary material for: miR-17-92 facilitates neuronal differentiation of transplanted neural stem/precursor cells under neuroinflammatory conditions
Source: J Neuroinflammation. 2016 Aug 27;13(1):208. doi: 10.1186/s12974-016-0685-5 (PMC5002215; doi:10.1186/s12974-016-0685-5)
Supplement: Additional file 1: Figures S1–S5. — Figure S1. Characterization of the NSCs. Figure S2. Effect of LIF and CNTF on differentiation of NSCs. Figure S3. miR-17-92 members directly target the 3′UTR of CNTFR or GP130. Figure S4. Bioinformatics analysis of miR-17-92 cluster members binding sites within CNTFR, GP130, JAK2, and STAT3. Figure S5. Traumatic brain injury. (PDF 558 kb) [file 12974_2016_685_MOESM1_ESM.pdf]

Figure S1

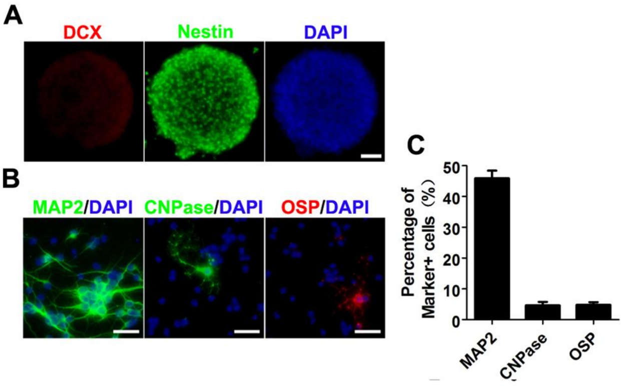

Figure S1. Characterization of the NSCs.

(A) Immunofluorescence labeling of whole-mount embryonic cortical neurospheres showing expression of DCX (red) and Nestin (green), DAPI (blue) was used for nuclear staining. Scale bar, 50 µm.

(B) The dissociated neurospheres were exposed to DMEM/F12/1%N2 plus media supplement medium to differentiate for 4 days in the absence of bFGF and hEGF followed by fluorescence staining with MAP2 (green), CNPase (green), oligodendrocyte specific protein (OSP) (red), DAPI (blue) was used for nuclear staining. Scale bar, 50 µm.

(C) The proportions of MAP2-positive, CNPase-positive and OSP-positive cells from the experiment shown in (B) were determined. Values are means ± s.e.m.

Figure S2

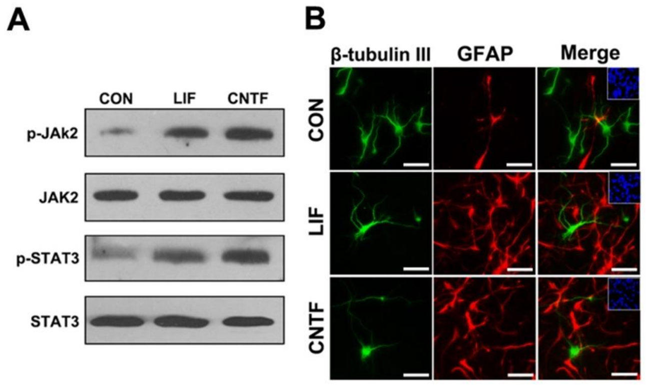

Figure S2. Effect of LIF and CNTF on differentiation of NSCs.

Dissociated NSCs were cultured for 48 h on poly-L-ornithine and human Fibronectin coated coverslips, and then treated with 10 ng/ml LIF or CNTF for 4 h. The cells were then cultured for another 48 h in the absence of bFGF and hEGF, and subjected to western blot analysis (A); or immunofluorescent staining (B), inset: DAPI staining of the cell nuclei in the field of view. Scale bar, 50 μm.

Figure S3

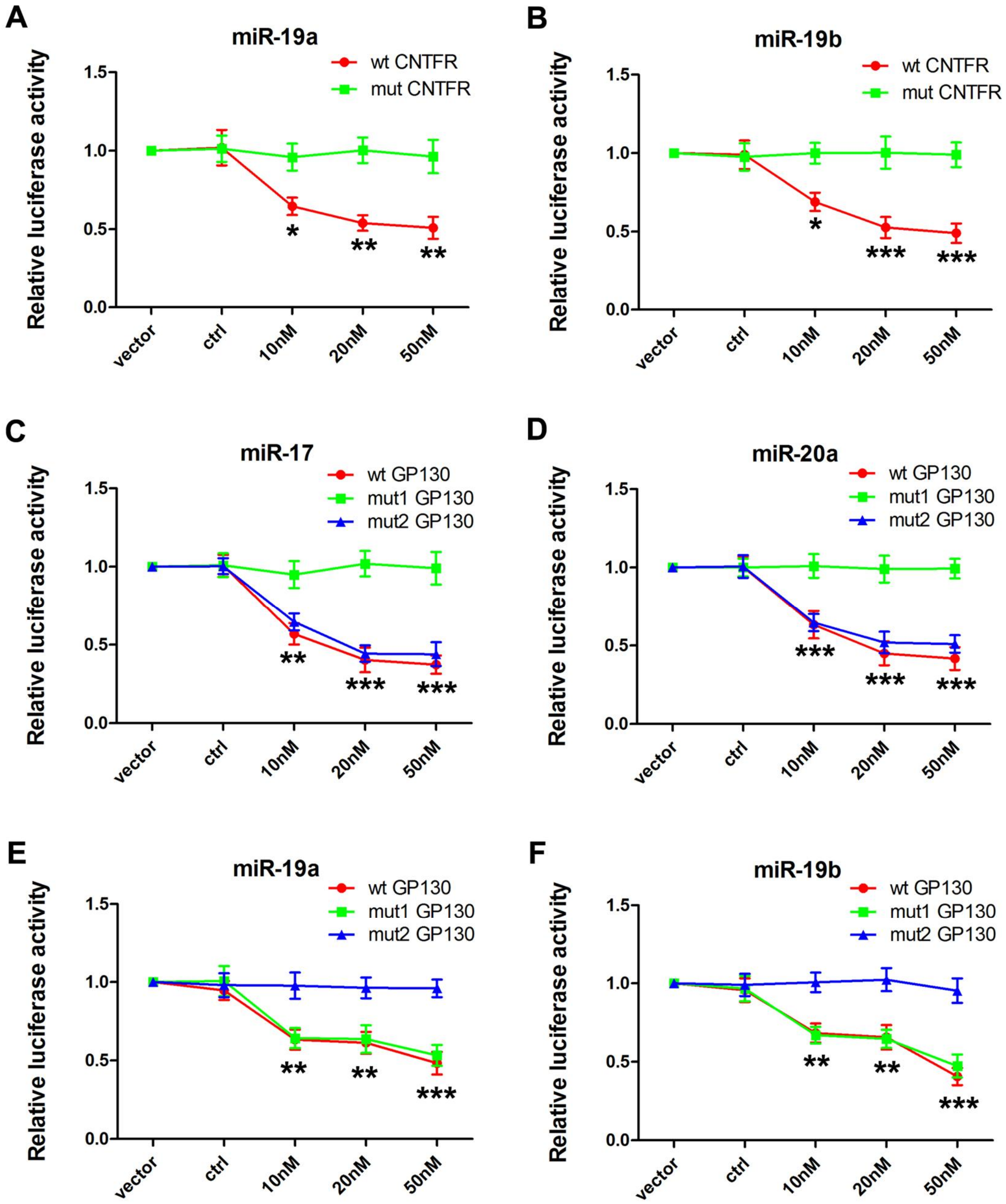

Figure S3. MiR-17-92 members directly target the 3'UTR of CNTFR or GP130.

Luciferase activity was measured 24 h after transfecting HEK 293T cells. Reporter plasmids with the wild-type (wt CNTFR, wt GP130) or mutated (mut CNTFR, mut1 GP130 or mut2 GP130) 3' UTR of CNTFR or GP130 were transfected either alone (vector) or with miR-17, miR-20a, miR-19a, miR-19b (10nM, 20nM or 50nM) or a scramble miRNA mimics (con). Values are means

± s.e.m. (*P < 0.05, **P < 0.01, ***P < 0.001, two-way ANOVA followed by Bonferroni post-test, between wild type and mutants, n = 3 independent experiments).

Figure S4

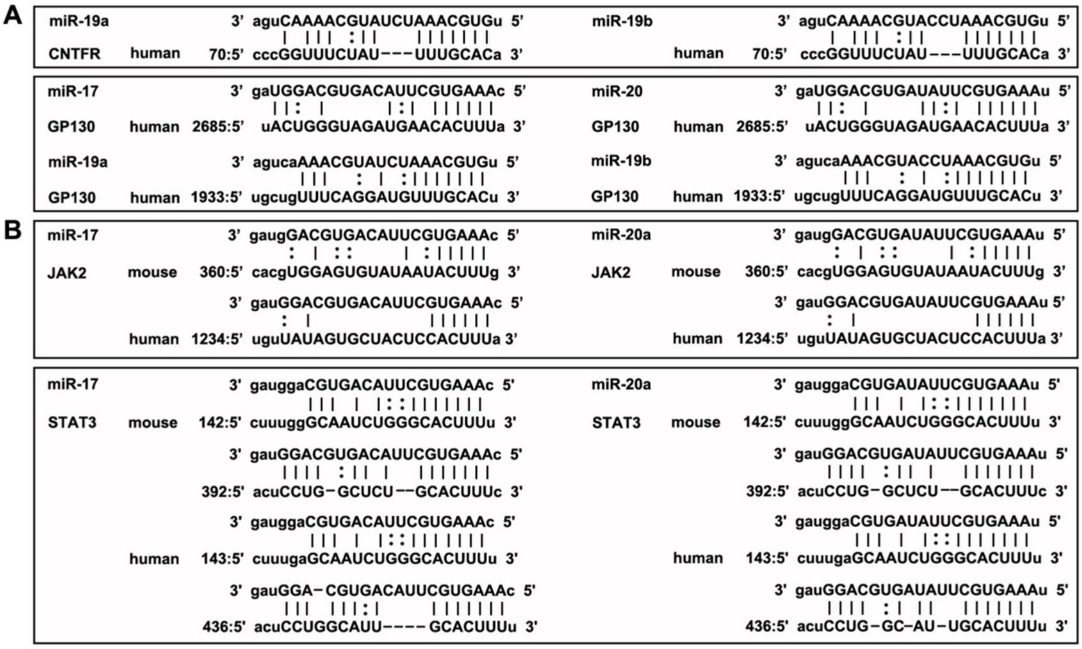

Figure S4. Bioinformatics analysis of miR-17-92 cluster members binding sites within CNTFR, GP130, JAK2 and STAT3.

(A) Predicted targeting sites of miR-17-92 cluster members within the 3'UTR of CNTFR and GP130 in human. (B) Predicted targeting sites of miR-17-92 cluster members within the 3'UTR of JAK2 and STAT3 in mouse and human.

Figure S5

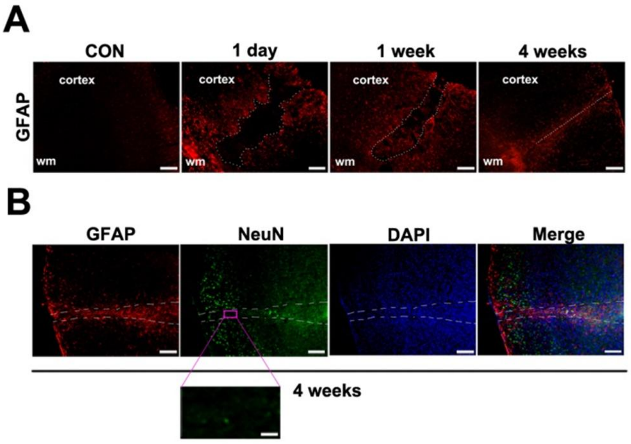

Figure S5. Traumatic brain injury.

(A) GFAP expression levels (red) around the injury site (between the white dotted lines) over time: 1 day, 1 week and 4 weeks after injury. The nucleus was stained with DAPI (blue). Scale bar, 200 µm. wm, white matter. (B) Immunofluorescence labeling of coronal sections for GFAP (red) and NeuN (green) 4 weeks after injury. DAPI (blue) was used for nuclear staining. Scale bar, 200 µm. The framed areas in (B) are shown at higher magnification. Scale bar, 25 µm.
